# Evaluation of the Accuracy and Intraprocedural Use of a Holographic Display for 3-Dimensional Transesophageal Echocardiography

**DOI:** 10.1016/j.cjco.2025.03.014

**Published:** 2025-03-24

**Authors:** Elchanan Bruckheimer, Alexander Lowenthal, Nili Schamroth Pravda, Hana Vaknin-Assa, Mordehay Vaturi, Gabriel Amir, Leor Perl, Pablo Codner, Yaron Shapira, Tamir Dagan, Ran Kornowski, Einat Birk

**Affiliations:** a Department of Pediatric Cardiology, Schneider Children’s Medical Center of Israel affiliated with the Faculty of Medicine, Tel Aviv University, Tel Aviv, Israel; b Department of Cardiology, Rabin Medical Center, Petach Tikva, affiliated with the Faculty of Medicine, Tel Aviv University, Tel Aviv, Israel

## Abstract

**Background:**

Real-time 3-dimensional transesophageal echocardiography (3DTEE) data acquired during interventional procedures are displayed on 2-dimensional screens, limiting intuitive depth perception and spatial comprehension. The study objectives were to evaluate the feasibility of intraprocedural use of a holography display during structural cardiology procedures employing 3DTEE, and to assess the accuracy of offline linear measurements in 3DTEE datasets.

**Methods:**

A prospective single-center study was conducted using the HOLOSCOPE-i to guide catheter-based procedures using intraprocedural 3DTEE. Qualitative measures of recognition of anatomic structures, 3D spatial comprehension, and interaction with intracardiac anatomic structures and catheter position were evaluated using a Likert scale. Additionally, a retrospective analysis of offline 3DTEE datasets of mitral valve measurements were performed using the HOLOSCOPE-i vs QLAB. Intra- and interobserver variability was assessed, assuming an intraclass correlation coefficient > 0.75 indicates adequate reliability.

**Results:**

A total of 13 patients were enrolled. In all cases, anatomic structures were identified in real time, and spatial comprehension was enhanced (Likert scale). No nausea or headache was reported by the operators. Retrospective analysis of 41 mitral valve 3DTEE datasets was performed. Annular diameter measurements (anteroposterior [AP] and anterolateral-posteromedial [AL-PM]) demonstrated a Pearson correlation of 0.89 (HOLOSCOPE-i) and 0.91 (QLAB). Intraobserver ICC for AP, AL-PM was 0.97, 0.94 (HOLOSCOPE-i) and 0.95, 0.97 (QLAB); interobserver ICC for AP and AL-PM was 0.77 and 0.88 (HOLOSCOPE-i) and 0.96 and 0.98 (QLAB).

**Conclusions:**

Holographic display of intraprocedural real-time 3DTEE data is feasible and augments the experience of the operator. Linear measurements in the 3D holographic display are accurate, with good correlation to those using 2-dimensional multiplanar reconstruction 3DTEE software.

**Clinical Trial Registration:**

MOH_2021-09-13_010255.

## Résumé

**Contexte:**

Les données obtenues d’une échocardiographie transœsophagienne en 3D (ETO 3D) en temps réel pendant une intervention s’affichent en 2D sur les écrans, ce qui limite la perception de profondeur intuitive et la compréhension spatiale. Objectifs de l’étude : évaluer la faisabilité de l’utilisation peropératoire d’un affichage holographique pendant des interventions structurelles en cardiologie faisant appel à l’ETO 3D; évaluer l’exactitude des mesures linéaires autonomes dans les bases de données d’ETO 3D.

**Méthodologie:**

Étude monocentrique prospective utilisant le HOLOSCOPE-i pour guider les interventions par cathéter faisant appel à l’ETO 3D peropératoire. Des mesures qualitatives de reconnaissance des structures anatomiques, de la compréhension spatiale en 3D et de l’interaction avec des structures anatomiques intracardiaques et la position du cathéter ont été évaluées au moyen de l’échelle de Likert. De plus, une analyse rétrospective des données de mesures de la valve mitrale en ETO 3D autonome a été menée en comparant le HOLOSCOPE-i à QLAB. Des variabilités intra- et interobservatrices ont été réalisées en assumant un coefficient de corrélation intraclasse (CCI) supérieur à 0,75 pour indiquer une fiabilité adéquate.

Résultats : 13 patients ont été recrutés. Dans tous les cas, les structures anatomiques ont été repérées en temps réel, et la compréhension spatiale a été améliorée [échelle de Likert]. Aucune nausée ni céphalée n’a été signalée par les opérateurs. Une analyse rétrospective a été menée sur des données d’ETO 3D de 41 valves mitrales. Des mesures du diamètre annulaire (antéro-postérieur [AP] et antérolatéral-postéro-médial [AL-PM]) ont révélé une corrélation de Pearson de 0,89 (HOLOSCOPE-i) et de 0,91 (QLAB). Le CCI intra-observateur pour l’AP et l’AL-PM était 0,97 et 0,94 [HOLOSCOPE-i] ainsi que 0,95 et 0,97 [QLAB]; le CCI interobservateur pour l’AP et l’AL-PM était 0,77 et 0,88 [HOLOSCOPE-i] ainsi que 0,96 et 0,98 [QLAB].

**Conclusions:**

L’affichage holographique peropératoire des données d’ETO 3D en temps réel est faisable et rehausse l’expérience de l’opérateur. Les mesures linéaires dans les affichages holographiques 3D sont exactes et en bonne corrélation avec celles obtenues avec un logiciel d’ETO 3D de reconstruction multiplanaire 2D. N^o^ d’enregistrement de l’essai clinique : MOH_2021-09-13_010255

Accurate cardiac imaging is vital in all structural cardiac interventional procedures.^1^^,^^2^ With the increasing complexity of these interventions, complete visualization, afforded by 3-dimensional (3D) imaging, has become paramount to comprehend the complex, dynamic interrelationships of anatomic structures and interventional tools.^3^^,^^4^ 3D transesophageal echocardiography (3DTEE) has evolved into an indispensable modality for imaging static and dynamic cardiac structures, facilitating the treatment of patients during percutaneous structural heart procedures.^4^ However, the 3D aspect of this modality is limited to sonographic data acquisition, and the image generated from these 3D data is typically confined to a 2-dimensional (2D) screen. Depth is simulated for the observer by rendering techniques that afford a partial appreciation of spatial relationships^2^, ^3^, ^4^; however, true depth perception is not possible.^5^^,^^6^

Recently, the combined use of 3D imaging with novel digital optical technologies has enhanced the interventionalist’s spatial comprehension and ability to perform pre- and intraprocedural quantitative evaluations in the 3D image.^7^^,^^8^ However, these stereoscopic-based solutions create only an illusion of a 3D image with only 1 focal plane with which interaction is achieved by gesturing or pointers, and direct interaction with an image at close range is not possible. Computer-generated holography is a mixed-reality digital optical technology that creates holograms from patients’ 3D volumetric data sets.^5^ This process is analogous to a 3D printer that uses light to reproduce the image. The hologram is an exact visual replication of the image in all 3 physical dimensions, and unlike a stereoscopic-based image, it has all the depth cues, focal planes, and specific coordinates of the original data it represents. The integration of real-time 3DTEE datasets of cardiac structures into holographic technology creates a dynamic 3D spatially accurate representation as a distinct physical entity in real 3D space, and it provides the operator with an interactive 3D image akin to the surgeon’s hands holding the heart.^5^^,^^9^

We report on the following: (i) the feasibility of the intraprocedural use of holography for structural and valvular heart procedures in real-time; and (ii) the accuracy of intuitive linear offline measurements in volumetric 3DTEE datasets of the mitral valve in a holographic display, vs measurements performed on 2D multiplanar software.

## Methods

A single-centre clinical study was performed in 2 parts—a prospective arm to assess the feasibility of generating holographic images during structural cardiac interventional procedures, and a retrospective arm to assess the accuracy of offline linear measurements in 3DTEE datasets. The study was approved by the Rabin Medical Center Ethical Committee (IRB# 0459-20-RMC). One author (E.B) had full access to all the data in the study and takes full responsibility for its integrity and the data analysis.

### Holographic system

**Figure 1:**
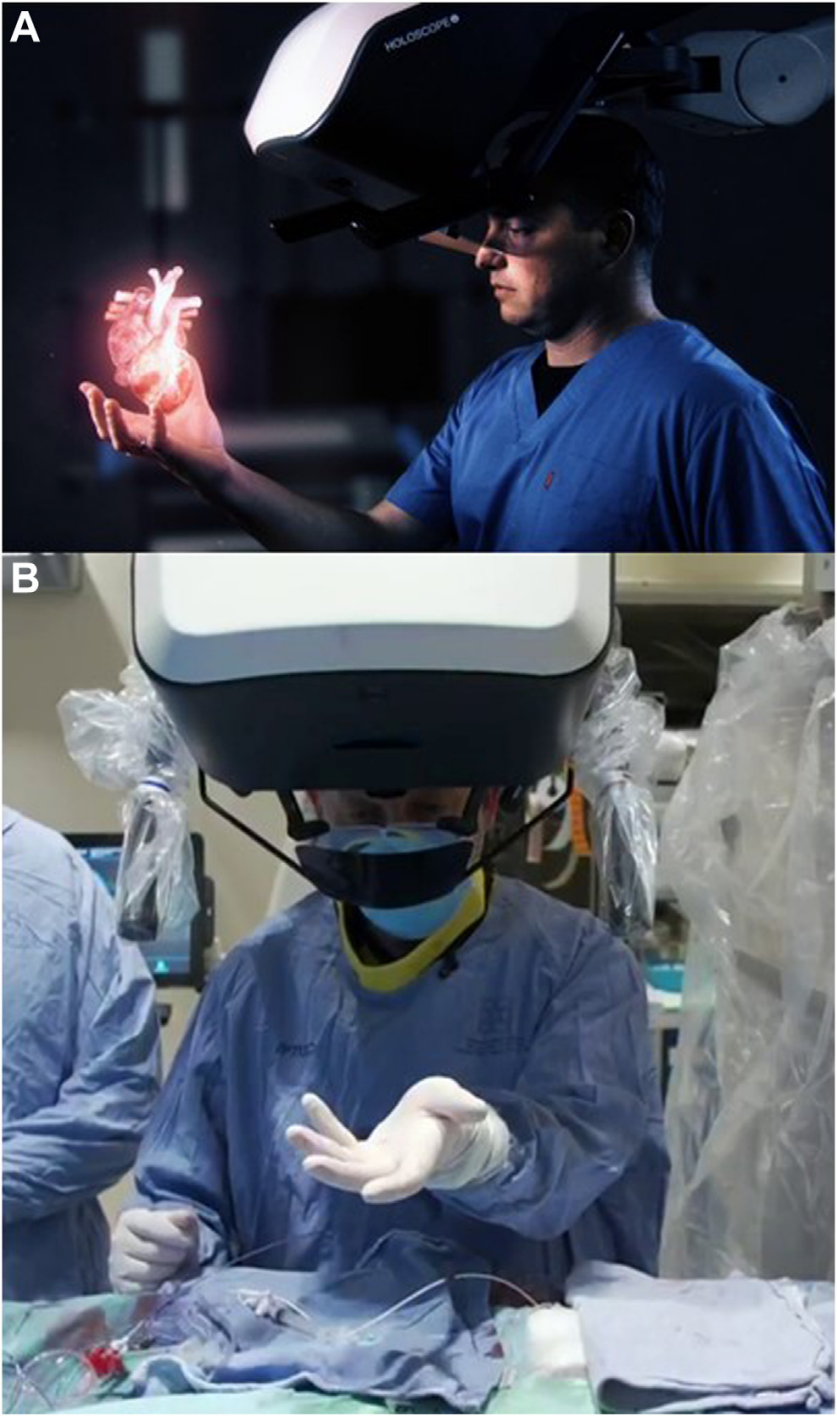
Interacting with the hologram viewed using the HOLOSCOPE-i (RealView Imaging, Yokneam, Israel; **A**) illustration and (**B**) actual. The interventional cardiologist sees the live hologram of the real-time 3-dimensional transesophageal echocardiography data floating in the air above the patient. Interactions are performed directly within the live image, including rotation, slicing, marking, and measurements.

The HOLOSCOPE-i (RealView Imaging, Yokneam, Israel) is a medical holographic display system approved by the US Food and Drug Administration that generates an online, live, dynamic, colour 3D hologram from real-time 3DTEE data, including colour Doppler. The viewer looks through a transparent eyepiece below the optical unit (Fig. 1) and directly interacts “in-air” with the image, by either finger movements, vocal commands (eg, rotate, slice, zoom), or by using a 3D mouse. Measurements are performed by directly placing 2 measurement points within the image, which can be manipulated simultaneously to precisely position these points at the desired plane or orientation.

### Intraprocedural use

Qualitative measures of the real-time intraprocedural use of the HOLOSCOPE-i system using real-time 3DTEE data were made by the performing interventional cardiologist, using a 4-point Likert scale (Supplemental Table S1). The following 4 parameters were assessed: visualization of anatomic structures; spatial comprehension of these structures; catheter-device orientation; and the added value of the system. The Likert scale is a validated rating score used to quantify a subjective level of agreement.^10^ Patients undergoing elective structural heart procedures using 3DTEE guidance were enrolled after they provided informed consent. The examinations were performed on an EPIQ CVX version 5.0.2 (Philips Medical Systems, Andover, MA) with an X8-2t transesophageal probe.

### Measurement accuracy

3DTEE datasets of adults who underwent assessment of native mitral valves at our institution between July 2021 and September 2021 were retrospectively evaluated offline. Linear measurements performed in these 3DTEE datasets on the HOLOSCOPE-i, and their intra- and interobserver variability, were compared to similar measurements performed on QLAB 13.0 software (Philips Medical Systems).

### Image selection and investigators

Datasets were selected according to eligibility criteria and allocated to normal and pathologic subgroups by a senior cardiologist. Images were uploaded in a random order, and measurements were performed by senior cardiologists trained on HOLOSCOPE-i and the QLAB MVN Model. All 41 sets were evaluated by one cardiologist on both displays and then repeated on a subset of 10 randomly selected datasets to assess intraobserver variability. The second cardiologist performed the measurements on the same subset on both displays to assess interobserver variability. The evaluators were blinded to the measurements of the comparative system and to each other.

### 3D measurements using QLAB MVN

MVN is semiautomated commercially available software (QLAB), for offline analysis of the mitral valve, that generates 3D models from 3DTEE datasets that are presented in 2D multiplanar reconstructions that can be edited manually to accurately assess the leaflets and annulus. The measurements used for the comparative study were the annular diameters (anteroposterior [AP] and anterolateral-posteromedial [AL-PM]), and these were made as previously described.^8^^,^^11^^,^^12^

### HOLOSCOPE-i measurements

**Figure 2:**
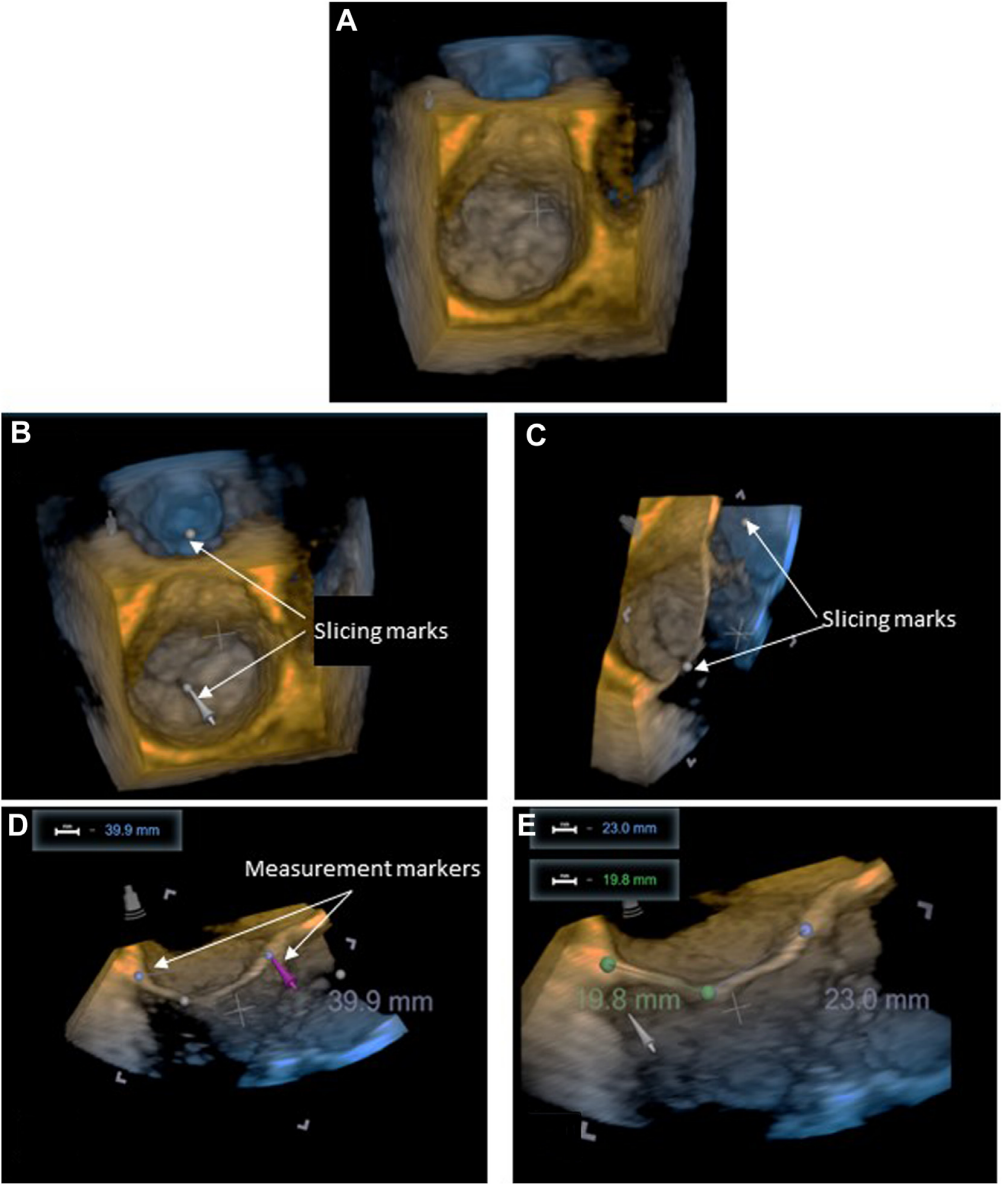
Main steps to measure mitral valve annulus and leaflets using the HOLOSCOPE-i (RealView Imaging, Yokneam, Israel). Pictures shown are as displayed on the 2-dimensional technician station, for explanation purposes only. (**A**) Transesophageal echocardiography–based full hologram, including the mitral valve. (**B**) anteroposterior (AP) plane marking; (**C**) AP slicing plane; and (**D**) AP linear measurement. (**E**) The anterior middle segment of mitral valve (A2), posterior middle sefment of mitral valve (P2) linear measurement.

The 3DTEE mitral valve datasets were imported manually into the HOLOSCOPE-i in Cartesian DICOM (Digital Imaging and Communications in Medicine) format and displayed as holograms. An end-systolic frame was selected, and the image was rotated to an *en face* view. Two points were placed—one at the perceived centre of the mitral valve, and the second along an imaginary line, to define the slicing plane (Fig. 2). The plane allowed for measuring the desired length by placing 2 markers at the annular edge while the image was cycled through systole and diastole to determine the exact position for leaflet insertion. A demostrative video of this is shown in Video 1(view video online).

### Study endpoints

The primary endpoint was concordance in measurements performed on the HOLOSCOPE-i, vs those performed on QLAB, and the secondary endpoints were the intraobserver and interobserver variability in measurements performed on the HOLOSCOPE-i vs those performed on QLAB. An additional goal of the study was to assess the technical success and safety of the system in providing real-time 3DTEE-based holographic imaging throughout the course of the procedures.

### Statistical analysis

Sample size assessment was performed assuming an intraclass correlation coefficient (ICC) > 0.75 to indicate good reliability, with a power of 80% and an alpha of 0.05.^13^ A minimum sample size of 36 patients was found to be sufficient for meeting the primary study endpoint, and a sample of 8 patients was found to be adequate for the secondary study endpoint (PASS software (for power analysis and sample size), version 19.0.4, NCSS, Kaysville, UT). To account for possible dropouts, sample sizes of 41 and 10 for the primary and secondary endpoints, respectively, were determined for this study. Comparisons between results of HOLOSCOPE-i and QLAB were performed for each measurement, for normal and pathologic valves. The ICC, the Pearson correlation coefficient (*r*), and Cronbach’s alpha were calculated on each subset of data. The intraobserver and interobserver ICC values were calculated for each measurement made for pathologic and normal valves for HOLOSCOPE-i and QLAB. Bland-Altman plots were used to compare the results of HOLOSCOPE-i and QLAB related to the 2 main features—AL-PM and AP, by valve status and overall. Statistical analyses were performed using JMP Pro Statistical Discovery software, version 16.0.0 (SAS Institute, Cary, NC).

## Results

### Real-time intraprocedural use of the HOLOSCOPE-i system

A total of 13 patients (7 male, 6 female) with a median age of 71.5 years (range: 6.1-76.2) were enrolled in the study. The structural heart procedures were atrial septal defect closure (n = 4), transcatheter edge-to-edge mitral valve repair (n = 5), left atrial appendage closure (n = 2), tricuspid valve edge-to-edge repair (n = 1), and paravalvular leak closure (n = 1). All 13 procedures were performed successfully and without complications. The HOLOSCOPE-i was used in the regular clinical workflow when 3DTEE imaging was used. Intraprocedural scores were reported by the operators at the end of each procedure and are depicted in Table 1. The scores were high for visualization, 3D spatial comprehension, and relationship of patient anatomy to catheter positioning. The use of the HOLOSCOPE-i was intuitive and straightforward for the operators.

**Table 1:** Intraprocedural evaluation

Feature	Likert score
Visualization	3.7 ± 0.5
Spatial comprehension	3.7 ± 0.5
Catheter-device orientation	3.8 ± 0.4
Added value	3.1 ± 0.9

Values are mean **±** standard deviation.

No adverse effects were reported by any of the investigators during the use of the HOLOSCOPE-i—specifically, no nausea or headache.

### Accuracy of measurements on the HOLOSCOPE-i system from 3DTEE datasets

Linear measurements of AP and AL-PM annular diameters in the 41 patient datasets (19 with normal valves, 22 with pathologic valves), performed retrospectively and offline, are summarized in Table 2. Overall, the correlation values are high, with correlation values being slightly higher for the pathologic subset.

**Table 2:** Correlations of measurements of mitral valve (MV) diameters between HOLOSCOPE-i (RealView Imaging, Yokneam, Israel) and QLAB (Philips Medical Systems, Andover, MA)

All MV diameters	Pearson correlation	Lower 95%	Upper 95%	Cronbach α
**AP**	0.89	0.81	0.94	0.94
**AL-PM**	0.91	0.83	0.95	0.95
**Normal MV diameters**				
**AP**	0.84	0.62	0.97	0.91
**AL-PM**	0.86	0.67	0.95	0.92
**Pathologic MV diameters**				
**AP**	0.94	0.87	0.98	0.97
**AL-PM**	0.93	0.85	0.97	0.97

AL-PM, anterolateral posteromedial; AP, anteroposterior.

### Intraobserver and interobserver variability

Intraobserver and interobserver variability data are summarized in Table 3. On a sample of 10 randomly selected images, the intraobserver agreement for AP and AL-PM measurements was high for both modalities, whether for normal or pathologic valves. The interobserver agreement was high for the AP and AL-PM measurements made with QLAB; it was less consistent for HOLOSCOPE-i, although still higher than the prespecified target of 0.75.

**Table 3:** Intra- and interobserver variability for normal and pathologic mitral valve (MV) on HOLOSCOPE-i (RealView Imaging, Yokneam, Israel) and QLAB (Philips Medical Systems, Andover, MA)

Intraobserver variability	ICC, QLAB		ICC, HOLOSCOPE-i	
	All MV		All MV	
**AP**	0.95		0.97	
**AL-PM**	0.97		0.94	
	Normal	Pathologic	Normal	Pathologic
**AP**	0.95	0.92	0.99	0.90
**AL-PM**	0.99	0.93	0.84	0.99
**Interobserver variability**	**ICC, QLAB**		**ICC,HOLOSCOPE-i**	
	**All MV**		**All MV**	
**AP**	0.96		0.77	
**AL-PM**	0.98		0.88	
	Normal	Pathologic	Normal	Pathologic
**AP**	0.97	0.95	0.80	0.75
**AL-PM**	0.99	0.96	0.91	0.86

AL-PM, anterolateral posteromedial; AP, anteroposterior; ICC, intraclass correlation coefficient.

### Bland-Altman plots

**Figure 3:**
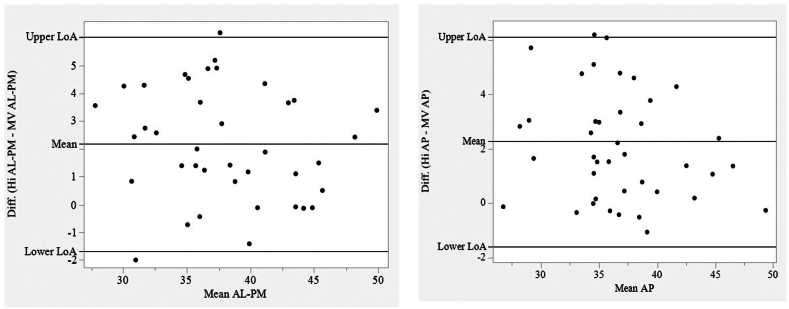
Bland-Altman plots of linear measurements**:** upper and lower limits of agreement (LoA) are calculated as the mean difference +/- 1.96∗standard deviation of the differences (Diff). The plots demonstrate no evidence of nonrandom patterns regarding differences between paired results of HOLOSCOPE-i (Hi; RealView Imaging, Yokneam, Israel) and QLAB (Philips Medical Systems, Andover, MA) measurements. AL-PM, anterolateral posteromedial; AP, anteroposterior; MV, mitral valve.

Bland-Altman plots were constructed for the linear measurements (Fig. 3). The plots demonstrate no evidence of nonrandom patterns regarding differences between the paired HOLOSCOPE-i and QLAB measurements.

## Discussion

This first-in-human study of the HOLOSCOPE-i demonstrated the following: (i) the accuracy of intuitive linear measurements of the mitral valve within the holographic image; and (ii) the feasibility of the intraprocedural use of real-time holography in real-world cardiac percutaneous interventions guided by 3DTEE. Linear measurements in 3D images displayed on 2D screens are inaccurate due to the parallax error, which occurs because of the lack of a z-axis, depth perception, and the fact that the markers cannot be placed accurately at a specific x,y,z coordinate of the image.^14^ Accurate measurements therefore require the generation of orthogonal 2D slices (xy, xz, yz) presented as a multiplanar reconstruction.^11^^,^^14^^,^^15^ In this study, we report a good correlation (≥ 0.75, Pearson, ICC] of freehand, intuitive, 3D linear measurements of the mitral valve in the hologram generated by HOLOSCOPE-i to those performed in the semiautomatic 2D program of MVN QLAB. This finding was predictable and met our hypothesis, as in a similar fashion, the accuracy of 3DTEE datasets was assessed by comparing the surgeon’s 3D anatomic mitral measurements to those made on QLAB as multiplanar reconstructed images of those datasets.^11^ The interobserver correlation for HOLOSCOPE-i was > 0.75 but it was less than the intraobserver correlation, due to the intuitiveness of the measurements. The annulus-leaflet margin is defined solely by manually placed markers in the HOLOSCOPE-i, whereas QLAB employs inherent automatic settings that directly minimize interobserver variation. Additional factors include the variability of pathologic leaflets and the influence of volume rendering on "tissue thickness." For these reasons, a small bias is observed on the Bland-Altman plots, although these are not clinically significant to the overall size of the valve. In a recent report of a comparative study of 3DTEE mitral valve measurements on QLAB and Echopixel (EchoPixel, Inc., San Jose, CA), a stereoscopic display of 3D datasets, a good overall correlation was achieved, but the interobserver correlation was < 0.75 for the linear measurements of AP and AL-PM annular diameters on Echopixel.^8^ This low correlation level may be related in part to the fact that the stereoscopic image has only 1 focal plane and that measurements are made by gesturing as opposed to direct interaction with the image voxels.

The good correlation of the linear measurements reported in our study emphasizes the fidelity and true depth perception provided by the HOLOSCOPE-i holographic images. This finding supports the clinical purpose of a real-time 3D holographic display, which is to provide an intuitive spatial comprehension of the dynamic interrelationships of the patient-specific anatomy and the catheters and devices that are used in structural heart procedures.

Methodologies that have been used to enhance the interventionalist’s comprehension include 3D printing^16^^,^^17^ and digital optical technologies.^7^^,^^9^ 3D-printed models are not instantaneous, and their applicability is limited to pre-procedure planning. Model manipulation, such as slicing, is somewhat difficult and typically is destructive. Digital optical technologies used in the catheterization laboratory include augmented reality and mixed reality displays, which import images into the environment, allowing the observer the ability to interact simultaneously with the environment and the digital overlay.^7^ Augmented reality and mixed reality create an illusion of 3-dimensionality and depth using stereoscopy, which mimics stereopsis, the different angulation of an object as viewed by either eye.^9^ The overlapping of the 2 versions of an image is close to the observer, whose eyesight converge to this point, but the focus on the image source is at a distance and therefore vergence and accommodation (focus) are uncoupled, often resulting in discomfort or nausea.^9^^,^^18^ Moreover, when attempting to touch the up-close stereoscopic image, the eyes converge to focus on the observer’s hand and lose their focus on the distant source of the image, which becomes blurred. Although stereoscopic displays, such as Echopixel, can generate high-quality 3D images, the inability to interact directly with the image, and the vergence–accommodation conflict, limits the intraprocedural use of this display. Computer generated holography is a mixed-reality digital optical technology that creates an exact visual replication of all the depth cues, focal planes, and specific coordinates of the original object or 3D image it represents. We previously reported the feasibility of creating holograms in real time in a cardiac catheterization laboratory using an early prototype.^5^ Based on these initial studies, we developed and designed the HOLOSCOPE-i to conform to the regular workflow in the current catheterization laboratory, thereby affording operators immediate access to an image of appropriate dimension and definition so they can interact with it freely, with no need to wear equipment.

**Figure 4:**
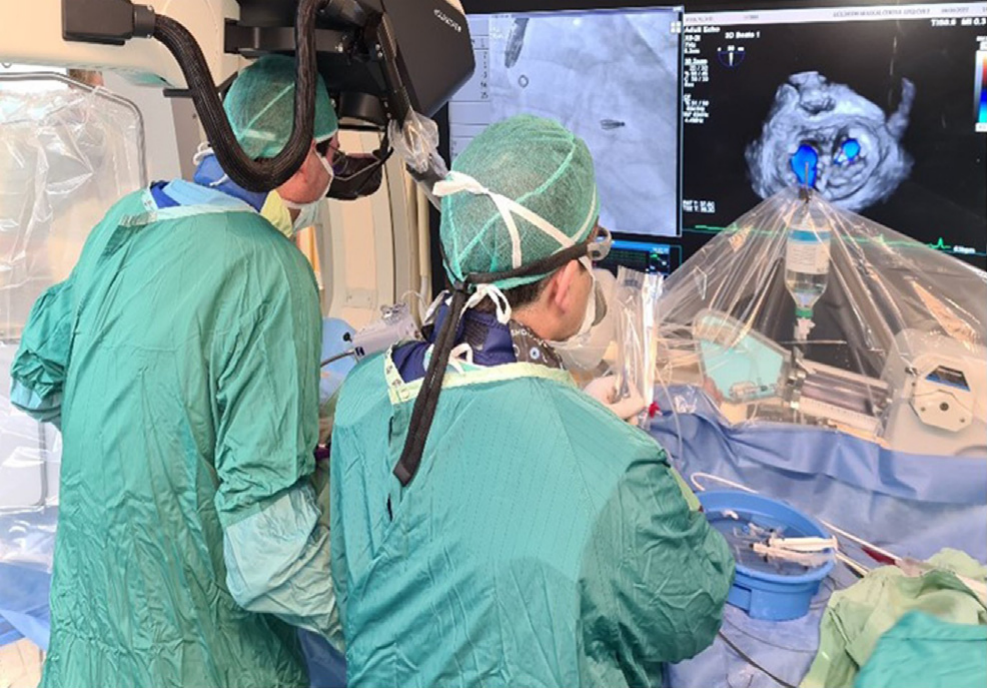
Intraprocedural use of HOLOSCOPE-i (RealView Imaging, Yokneam, Israel) during a mitral valve intervention guided by 3-dimensional transesophageal echocardiography.

The HOLOSCOPE-i was designed to conform to the regular workflow in the current catheterization laboratory. The optical unit is situated high above the user’s head and is pulled down only when needed; the hologram is viewed floating in free space, allowing the user to touch the image in a sterile manner while performing the procedure (Fig. 4). In addition, studies were performed to prove the fidelity of the holographic image to the data acquired from the imaging modality, among other requirements, to achieve regulatory approval. In the current study, the high score on the Likert scale demonstrates that the intraprocedural use of the HOLOSCOPE-i for structural heart interventions guided by 3DTEE was feasible, intuitive, and safe, afforded identification of all anatomic structures, and improved 3D spatial comprehension of the interrelationship of cardiac structures, catheters, and devices during the procedure. These novel features were noted particularly in the atrioventricular clip implantation procedures, for which free 3D navigation to a specific moving target on the mitral and tricuspid valve leaflets can be demanding. This ability to directly interact with the patient's real-time 3-dimensional anatomy, as if holding the heart in one's hands, might have a significant impact on the performance, procedure time, and outcome of minimally invasive interventions, and this needs to be evaluated in appropriately designed and controlled clinical trials.

This study has some limitations. The use of intraprocedural holography was evaluated on a small number of selected patients by experienced interventional and imaging cardiologists, and the clinical impact requires further validation in future trials. The limited sample size reflects the preliminary nature of this work and the practical constraints of studying a novel technology in real-world setting. The small sample size limits the statistical power and generalizability of the results. The study provides preliminary findings and paves the way for larger, more detailed studies that can assess procedural variability and performance more comprehensively.

### Conclusions

Holographic display of intraprocedural, real-time 3DTEE data is feasible and enhances the intraprocedural experience of the operator. Linear measurements in the 3D holographic display are accurate and correlate well with those made using 2D multiplanar reconstruction 3DTEE software. Our findings are promising and encourage further investigation into the clinical benefits of this novel technology.

## Supplementary Material

Video This video demonstrates a hologram of a 3DTEE image of a dynamic mitral valve. The valve is rotated with the operator's finger directly touching the image and then using voice commands the image is sliced and enlarged. The measurement is made by placing markers intuitively on the image and when rotated it can be noted that the markers move with the image remaining where they were placed

## References

[1] Rat N., Muntean I., Opincariu D. (2020). Cardiovascular imaging for guiding interventional therapy in structural heart diseases. Curr Med Imaging Rev.

[2] Hahn R.T., Saric M., Faletra F.F. (2022). Recommended standards for the performance of transesophageal echocardiographic screening for structural heart intervention: from the American Society of Echocardiography. J Am Soc Echocardiogr.

[3] Ro R., Bamira D., Bernard S. (2023). Transesophageal echocardiographic screening for structural heart interventions. Curr Cardiol Rep.

[4] Wollborn J., Schuler A., Sheu R.D., Shook D.C., Nyman C.B. (2023). Real-time multiplanar reconstruction imaging using 3-dimensional transesophageal echocardiography in structural heart interventions. J Cardiothorac Vasc Anesth.

[5] Bruckheimer E., Rotschild C. (2016). Holography for imaging in structural heart disease. EuroIntervention.

[6] Barreiro-Perez M., Estévez-Loureiro R., Puga L. (2022). Real-time echocardiography-fluoroscopy fusion imaging with automated 3d heart segmentation during transcatheter structural heart interventions. JACC Cardiovasc Interv.

[7] Silva J.N.A., Southworth M., Raptis C., Silva J. (2018). Emerging applications of virtual reality in cardiovascular medicine. JACC Basic Transl Sci.

[8] Ballocca F., Meier L.M., Ladha K. (2019). Validation of quantitative 3-dimensional transesophageal echocardiography mitral valve analysis using stereoscopic display. J Cardiothorac Vasc Anesth.

[9] Bruckheimer E., Rotschild C., Dagan T. (2016). Computer-generated real-time digital holography: first time use in clinical medical imaging. Eur Heart J Cardiovasc Imaging.

[10] Sullivan G.M., Artino A.R. (2013). Analyzing and interpreting data from Likert-type scales. J Grad Med Educ.

[11] Biaggi P., Jedrzkiewicz S., Gruner C. (2012). Quantification of mitral valve anatomy by three-dimensional transesophageal echocardiography in mitral valve prolapse predicts surgical anatomy and the complexity of mitral valve repair. J Am Soc Echocardiogr.

[12] Saito K., Okura H., Watanabe N. (2011). Quantification of mitral valve apparatus by three-dimensional transesophageal echocardiography: in vitro validation study comparing two different analysis systems. J Echocardiogr.

[13] Koo T.K., Li M.Y. (2016). A guideline of selecting and reporting intraclass correlation coefficients for reliability research. J Chiropr Med.

[14] Mahmood F., Jeganathan J., Saraf R. (2016). A practical approach to an intraoperative three-dimensional transesophageal echocardiography examination. J Cardiothorac Vasc Anesth.

[15] Mashari A., Montealegre-Gallegos M., Knio Z. (2016). Making three-dimensional echocardiography more tangible: a workflow for three-dimensional printing with echocardiographic data. Echo Res Pract.

[16] Ooms J.F., Wang D.D., Rajani R. (2021). Computed tomography-derived 3D modeling to guide sizing and planning of transcatheter mitral valve interventions. JACC Cardiovasc Imaging.

[17] Wang D.D., Gheewala N., Shah R. (2018). Three-dimensional printing for planning of structural heart interventions. Interv Cardiol Clin.

[18] Lin C.J., Canny S. (2022). Effects of virtual target size, position, and parallax on vergence-accommodation conflict as estimated by actual gaze. Sci Rep.

