# Supplementary material for: Evaluation of the Accuracy and Intraprocedural Use of a Holographic Display for 3-Dimensional Transesophageal Echocardiography
Source: CJC Open. 2025 Mar 24;7(6):728–35. doi: 10.1016/j.cjco.2025.03.014 (PMC12198600; doi:10.1016/j.cjco.2025.03.014)
Supplement: Supplementary Table [file mmc2.docx]

Supplemental Table S1: Intraprocedural use of Holoscope-i: Likert Scale

| Visualization: The overall quality of image based on the ability to visualize the relevant structures for the procedure | |
| --- | --- |
| 1 | Structures cannot be identified |
| 2 | Some structures can be identified |
| 3 | All structures can be identified |
| 4 | All structures can be easily identified |
| Spatial comprehension: The overall quality of the image by assessing the spatial comprehension of the structures in the images | |
| 1 | 3D spatial relationships cannot be identified |
| 2 | Some 3D spatial relationships can be identified |
| 3 | All 3D spatial relationships can be identified |
| 4 | All 3D spatial relationships can be identified intuitively |
| Catheter-device orientation: Quality of image in providing orientation and placement comprehension of the tools used during the procedure in relation of the anatomical structures: | |
| 1 | Orientation and placement cannot be understood |
| 2 | Orientation and placement can be understood |
| 3 | Orientation and placement can be easily understood |
| 4 | Orientation and placement can be intuitively understood |
| Added value: Based on previous experience using 3D ultrasound on 2D screens does the use of the HOLOSCOPE-i provide added value in understanding the spatial anatomical relationships? | |
| 1 | Does not provide any added value |
| 2 | Provides some value |
| 3 | Provides additional value |
| 4 | Provides additional value for an intuitive understanding |
